# Comparative outcomes of cryoextraction and conventional extraction in anterior and lateral orbitotomy for orbital cavernous venous malformations (so-called cavernous hemangioma)

**DOI:** 10.1186/s12886-026-05111-1

**Published:** 2026-07-07

**Authors:** Joris De Keersmaecker, Luciano Accetta, David Muallah, Hendrik Schulz, Eckart Bertelmann

**Affiliations:** Department of Ophthalmology, Charité – Universitätsmedizin Berlin, Berlin, Germany

**Keywords:** Cavernous venous malformation, Cavernous hemangioma, Lateral orbitotomy, Swinging eyelid approach, Anterior orbitotomy, Cryoextraction, Surgical outcomes

## Abstract

**Background:**

Comparison of surgical outcomes of lateral orbitotomy and transconjunctival anterior orbitotomy using the swinging eyelid approach for cavernous venous malformations (CVM), with and without cryoextraction.

**Methods:**

In this single-center, retrospective case series (2005–2024), a single surgeon performed CVM excisions. Operative time and postoperative complications were compared across surgical approaches and extraction techniques for a cohort of 60 patients.

**Results:**

Lateral orbitotomy was performed in 25 patients (14 conventional, 11 cryo-assisted) and the swinging eyelid approach in 35 patients (16 conventional, 19 cryo-assisted). In the swinging eyelid group, cryoextraction significantly reduced operative time (49.0 vs. 59.0 min, *p* = 0.04). A similar trend was observed in the lateral orbitotomy group, although the difference was not statistically significant (81.0 vs. 101.5 min, *p* = 0.23). Postoperatively, visual acuity remained stable or improved in 97% of patients. Diplopia occurred less frequently following cryo-assisted extraction and was transient in most cases, with persistent diplopia requiring further intervention occurring exclusively after conventional extraction.

**Conclusions:**

Cryoextraction is associated with shorter operative times and favorable functional outcomes in the surgical management of orbital cavernous venous malformations, particularly when using the swinging eyelid approach. When applied in appropriately selected cases, cryoextraction represents a safe and effective technique that may reduce surgical manipulation and postoperative morbidity.

## Background

Cavernous venous malformation (CVM) of the orbit, formerly known as orbital cavernous hemangioma (OCH), is the most common benign orbital tumor in adults and a frequent reason for orbital surgery. Although histologically benign, its slow but progressive growth can cause functional and cosmetic morbidity, including proptosis, diplopia, and vision loss [1]. These lesions most frequently emerge in the fourth to fifth decade of life and are more common in women, possibly as a result of hormonal influences on tumor development [2, 3].

Most CVMs are located within the intraconal space, particularly lateral to the optic nerve [4, 5]. Multiple and bilateral cases are rare and may occur in syndromal contexts [6]. Histopathologically, they consist of dilated venous channels within a fibrous capsule, with hemosiderin deposits. The lack of arterial supply leads to vascular stasis, thrombosis, and subsequent stromal remodelling [7, 8].

Imaging is essential for diagnosis and surgical planning. Magnetic resonance imaging (MRI) is the preferred diagnostic modality due to its superior soft-tissue contrast and ability to delineate lesion–tissue relationships. Diffusion-weighted imaging (DWI) and apparent diffusion coefficient (ADC) values aid the differentiation from malignant lesions [7, 9–12].

Management ranges from observation for stable and asymptomatic lesions to surgical excision for symptomatic or enlarging masses. Surgical options include lateral orbitotomy and transconjunctival anterior orbitotomy using the swinging eyelid approach. The approach depends on lesion location, size, and relation to adjacent structures [13–15].

While the safety and feasibility of cryo-assisted surgery for orbital tumors have been reported [16–20], its potential to reduce operative time and improve postoperative outcomes remains unexplored. Notably, no study to date has evaluated whether cryoextraction influences surgery duration in both anterior and lateral orbitotomy for CVM removal. The present study compares surgery duration and postoperative outcomes between cryo-assisted and conventional extraction, both for lateral orbitotomy and transconjunctival anterior orbitotomy with the swinging eyelid approach. We propose that incorporating cryoextraction reduces surgery duration and improves postinterventional recovery.

## Methods

### Study design and ethics

In this retrospective, non-randomized, single-center study, data from 60 patients who underwent surgical removal of CVM were analyzed. For all patients the diagnosis of CVM was confirmed by histopathological examination of the excised tissue. All surgeries were performed from January 2005 to December 2024 at the Department of Ophthalmology, Charité - Universitätsmedizin Berlin, Germany.

The study protocol (trial registration EA4/067/24) was approved by the ethics committee of Charité - Universitätsmedizin Berlin, Germany. The data extraction and analysis was conducted according to the tenets of the Declaration of Helsinki.

### Data content and follow-up

The collected data included age, sex, affected side, symptoms, location and size of the mass, surgical technique (including whether cryoextraction was used), duration of surgery, and documentation of any postoperative complications.

All patients underwent standard pre- and postoperative eye examinations for orbital surgery. This included assessments of visual acuity (VA), Hertel/Naugle exophthalmometry, motility/diplopia examinations and pupillary light reflex examinations. Patients were examined daily during the first 2 to 3 days after surgery. Follow-up appointments were conducted after one week and one month.

Indications for surgery included symptomatic proptosis, diplopia, visual disturbance, documented tumor growth, compressive optic neuropathy, or cosmetically significant orbital asymmetry. Asymptomatic lesions detected incidentally on imaging were generally managed conservatively and were not included unless progressive enlargement or functional impairment prompted surgical intervention.

### Surgical technique

All surgeries were performed by the same experienced surgeon (E.B.). Conventional extraction techniques were used throughout the study period, whereas cryoextraction was introduced at our institution in 2016 and was increasingly adopted thereafter. The choice of extraction technique was made preoperatively based on the surgeon’s assessment of imaging findings and anticipated surgical complexity. Cryoextraction was preferentially considered in cases with radiological findings strongly suggestive of cavernous venous malformation and in lesions where conventional removal was expected to require more extensive dissection due to tumor location. In cases with less certain preoperative diagnosis, conventional extraction was more commonly performed. The increasing use of cryoextraction over time also reflected improvements in orbital imaging quality and growing surgical experience with the technique.

The choice of surgical approach was based on careful preoperative radiological evaluation, with consideration of tumor size and location in order to avoid crossing the optic nerve and to minimize its manipulation.

Transconjunctival anterior orbitotomy using a swinging eyelid approach was employed for CVMs located in the inferior orbit and intraconal space. The procedure was performed through an incision along the lower eyelid margin just below the lateral canthus, with transection of the inferior limb of the lateral palpebral ligament. The orbital septum was then opened via a conjunctival incision in the inferior fornix.

Lateral transosseous orbitotomy was used for lesions located in the lateral or superior orbit, as well as for tumors extending to the orbital apex. This approach was performed through a skin incision placed in the upper eyelid crease with lateral extension over the lateral orbital rim (Stallard–Wright approach). Following periosteal incision and careful subperiosteal dissection, the lateral orbital wall was temporarily removed and subsequently repositioned at the end of the procedure.

**Fig. 1:**
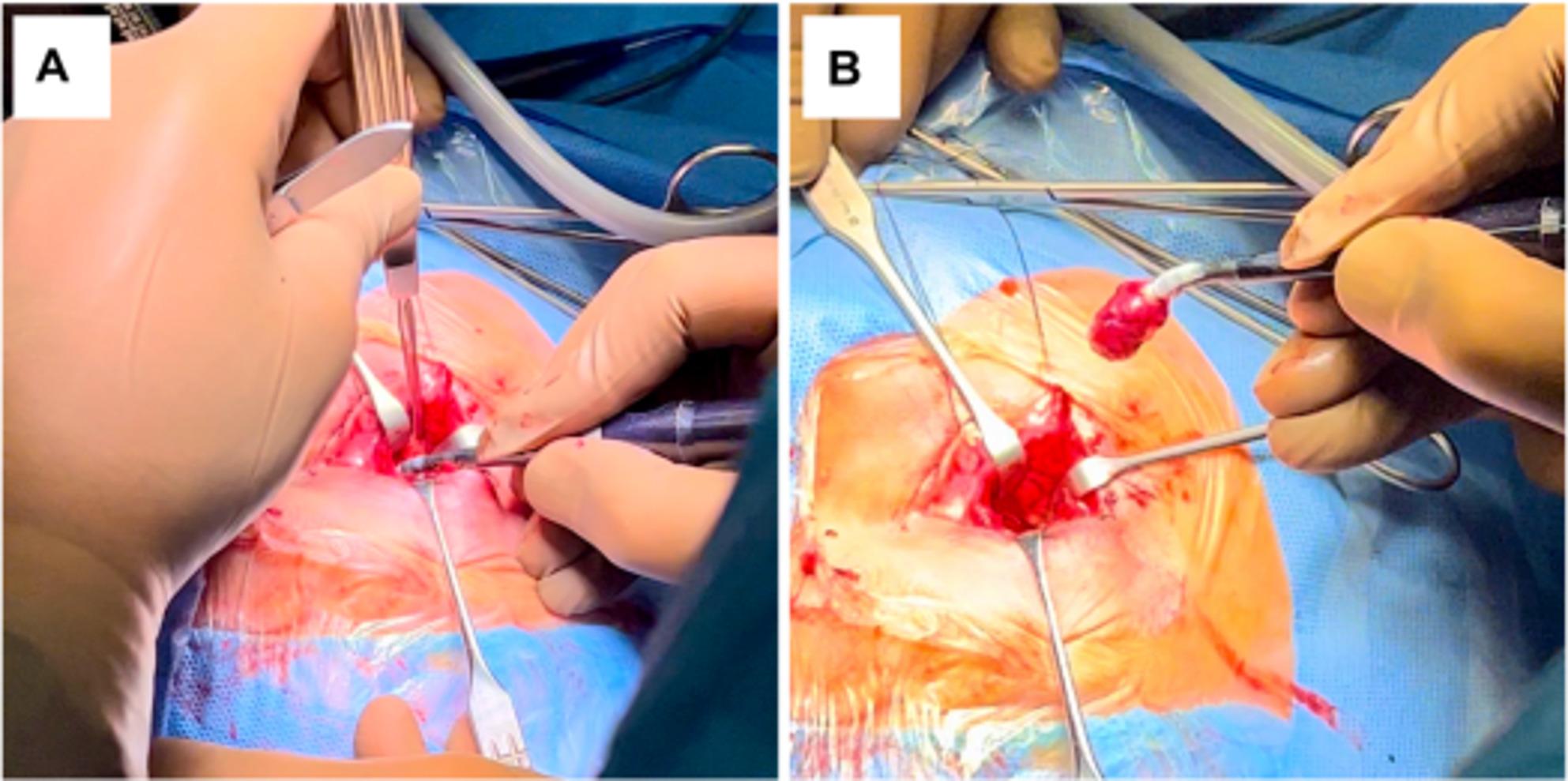
Clinical image of cryofixation of a CVM (**A**) and subsequent cryoextraction (**B**) from the left orbit through anterior orbitotomy with the swinging eyelid technique

Complete removal of the cavernous venous malformation was achieved in all patients. In conventional extraction, tumor delivery was performed using blunt dissection and traction with a 4 − 0 silk suture placed through the tumor mass. In cryo-assisted extraction, a cryoprobe was applied to the tumor surface to achieve fixation of the lesion, after which the mass was delivered using gentle traction while surrounding tissues were stabilized with a blunt spatula or elevator (Fig. 1).

### Statistical analysis

All statistical analyses were performed using RStudio (Version 2025.05.0) with the packages “ggplot2” for the plots and “gtsummary” for demographic tables. The distribution of surgery duration within each group was assessed visually using histograms. Although the lateral orbitotomy cohort appeared approximately normally distributed, the subgroup sample sizes were small. Therefore, non-parametric methods were used for both subgroup analyses. Surgery duration between conventional extraction and cryoextraction was compared using the Wilcoxon rank-sum test for both the lateral orbitotomy and the anterior orbitotomy with swinging eyelid approach.

## Results

### Patient demographics and baseline characteristics

Our retrospective analysis included all patients with a confirmed histopathological diagnosis of CVM that underwent surgery at our ophthalmological hospital between 2005 and 2024. Data from a total of 60 patients were retrospectively analyzed in this study, comprising 39 women (65%) and 21 men (35%) (Table 1). The median age at the time of surgery for the entire cohort was 55 years. Among all cases, 38 tumors (63%) were located intraconal, while 22 tumors (37%) were extraconal.

All the masses were removed using two types of surgical access: lateral orbitotomy (*n* = 25; 42%) and anterior orbitotomy with swinging eyelid approach (*n* = 35; 58%). Both approaches were performed either with or without cryoextraction. Of the 25 lateral orbitotomies, 14 cases (56%) were performed with conventional extraction and 11 (44%) with cryoextraction (Table 2). In the swinging eyelid group, 16 tumors (46%) were removed with conventional extraction and 19 (54%) with cryoextraction (Table 3).

Mean tumor sizes were as follows: 18.3 mm (conventional) and 17.6 mm (cryoextraction) for lateral orbitotomy; 15.6 mm (conventional) and 15.0 mm (cryoextraction) for the swinging eyelid approach. Each of these four subgroups included both intraconal and extraconal tumors.

**Table 1:** Patient demographics and baseline characteristics

Characteristic	Overall	Lateral orbitotomy	Swinging eyelid approach	*p*-value^2^
	*N* = 60^1^	*N* = 25^1^	*N* = 35^1^	
Age	55.0 (43.5, 64.5)	54.0 (42.0, 65.0)	56.0 (44.0, 64.0)	0.8
Sex				0.9
Male	21 (35%)	9 (36%)	12 (34%)	
Female	39 (65%)	16 (64%)	23 (66%)	
Surgery duration (min)	61.5 (49.0, 97.0)	92.0 (78.0, 119.0)	52 (41.0, 67.0)	< 0.001

^1^ Median (Q1, Q3); n (%)

^2^ Wilcoxon rank sum test; Pearson´s Chi-squared test

**Table 2:** Patient demographics for lateral orbitotomy

Characteristic	Overall	Conventional extraction	Cryoextraction	*p*-value^2^
	*N* = 25^1^	*N* = 14^1^	*N* = 11^1^	
Age	53.9 ± 20.1	51.9 ± 23.3	57.3 ± 15.6	0.6
Sex				0.2
Male	9 (36%)	7 (50%)	2 (18%)	
Female	16 (64%)	7 (50%)	9 (82%)	
Surgery duration (min)	92.0 (78.0, 119.0)	101.5 (85.0, 128.0)	81.0 (51.0, 119.0)	0.23

1 Median (Q1, Q3); n (%)

2 Wilcoxon rank sum test; Fisher´s exact test

**Table 3:** Patient demographics for anterior orbitotomies with swinging eyelid approach

Characteristic	Overall	Conventional extraction	Cryoextraction	*p*-value^2^
	*N* = 35^1^	*N* = 16^1^	*N* = 19^1^	
Age	56.0 (44.0, 64.0)	53.0 (40.5, 62.0)	58.0 (45.0, 64.0)	0.4
Sex				0.7
Male	12 (34%)	6 (38%)	6 (32%)	
Female	23 (66%)	10 (63%)	13 (68%)	
Surgery duration (min)	52.0 (41.0, 67.0)	59.0 (48.0, 96.5)	49.0 (35.0, 61.0)	**0.04**

^1^ Median (Q1, Q3); n (%)

^2^ Wilcoxon rank sum test; Pearson´s Chi-squared test

### Surgery duration

The median surgery duration across the full cohort was 61.5 min. Lateral orbitotomy procedures required significantly longer operative times than anterior orbitotomy with the swinging eyelid approach (92.0 vs. 52.0 min, *p* < 0.001).

**Fig. 2:**
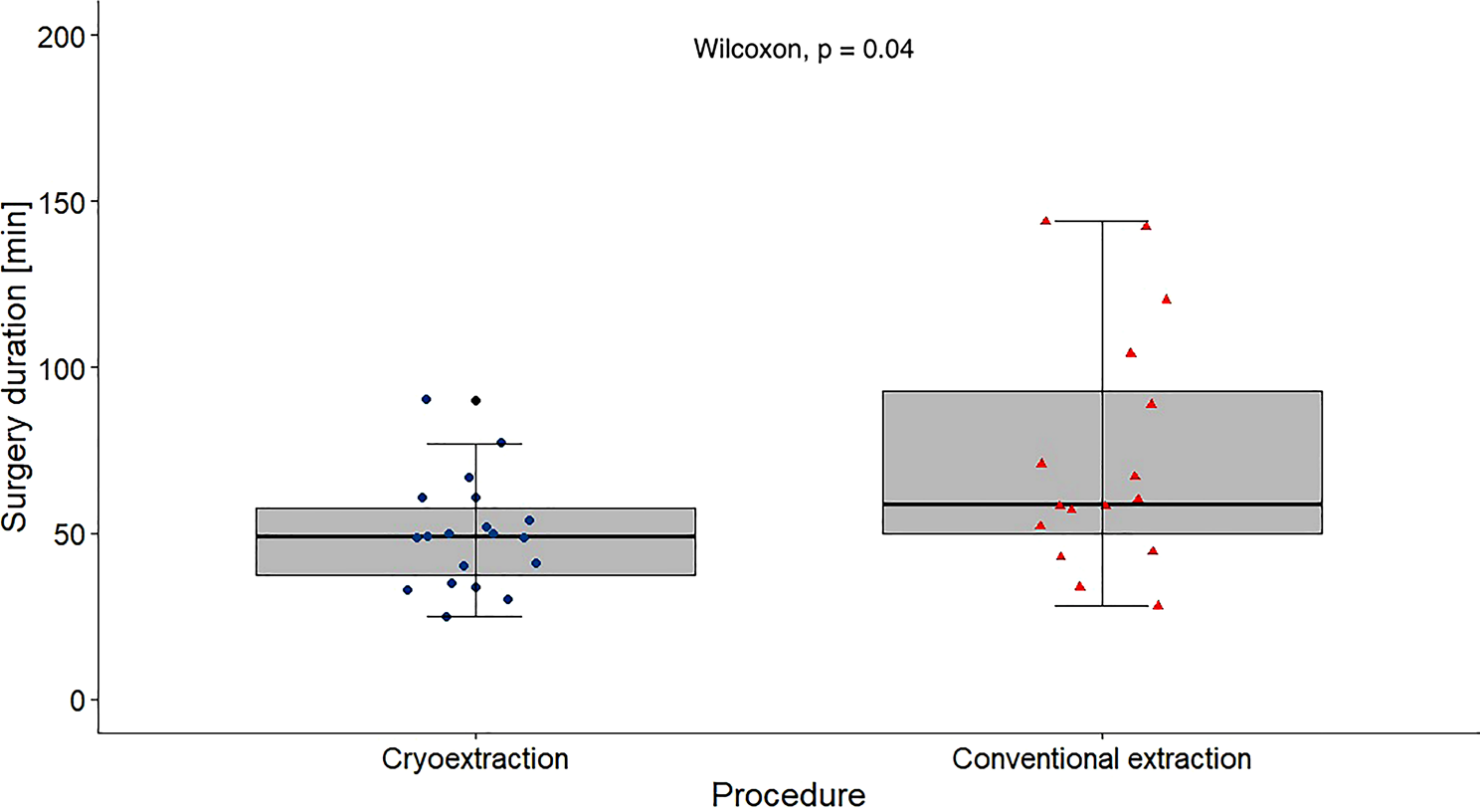
Comparison of surgery duration between cryoextraction (blue circles) vs. conventional extraction (red triangles) for anterior orbitotomy with swinging eyelid approach

**Fig. 3:**
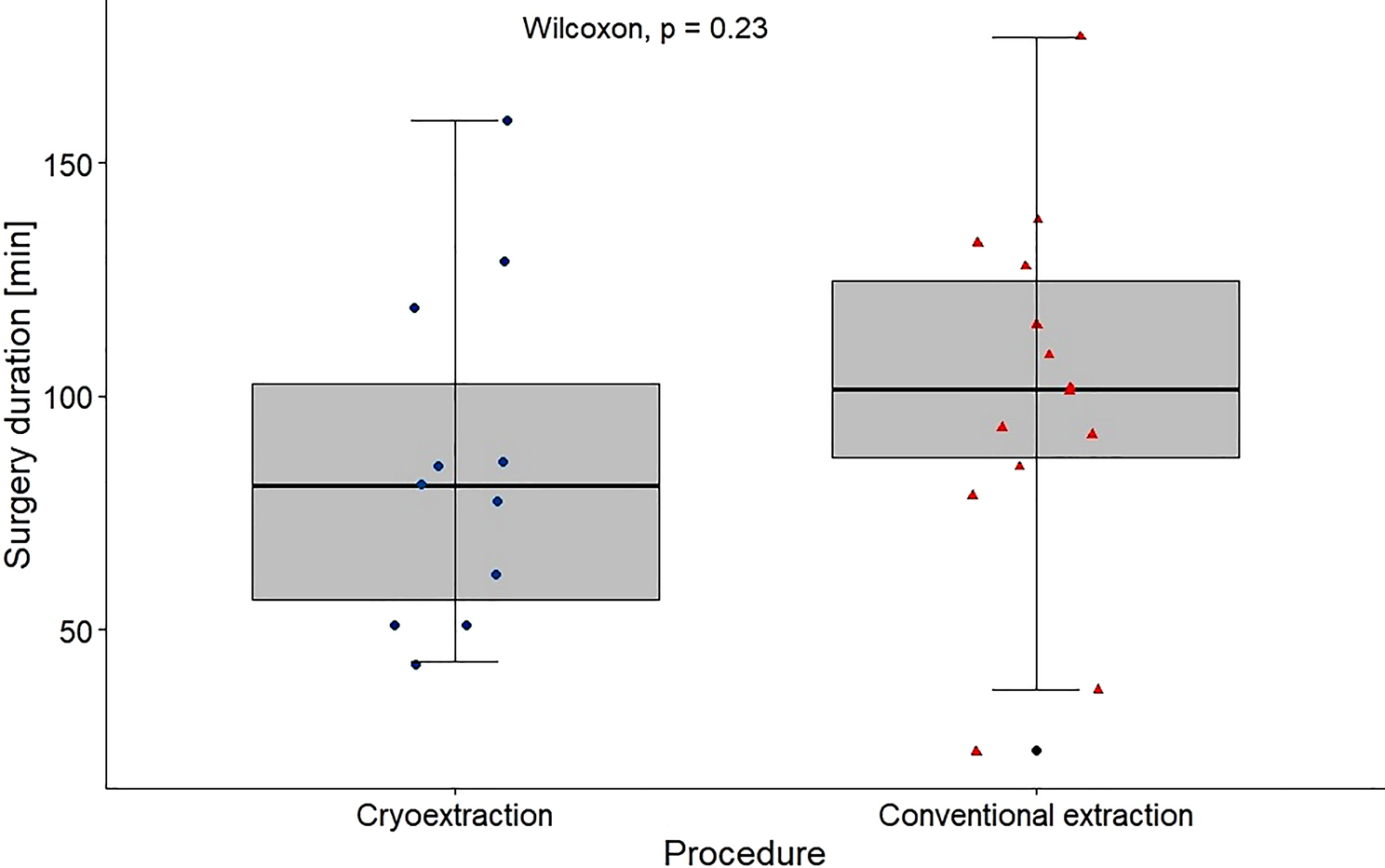
Comparison of surgery duration between cryoextraction (blue circles) vs. conventional extraction (red triangles) for lateral orbitotomy

Within the subgroup of 35 patients who underwent surgery using the swinging eyelid approach, operative times were significantly shorter with cryoextraction than with conventional extraction (49.0 vs. 59.0 min, *p* = 0.04; Fig. 2). A similar trend was observed in the lateral orbitotomy subgroup, with shorter operative times following cryoextraction (81.0 vs. 101.5 min, *p* = 0.23), although this difference did not reach statistical significance (Fig. 3).

### Functional outcomes and postoperative complications

Postoperatively, mean Snellen visual acuity remained stable overall, improving slightly from 0.86 preoperatively to 0.87 postoperatively. Mean Hertel exophthalmometry values decreased from 17.6 mm preoperatively to 15.9 mm postoperatively, reflecting reduction of orbital mass effect after surgery. Diplopia was present preoperatively in 20 patients (33%) and persisted postoperatively in 9 patients (15%), indicating an overall reduction in diplopia following surgery (Table 4).

Postoperatively, visual acuity improved in 24 patients (40%), remained stable in 34 (57%), and worsened in 2 (3%). One patient whose visual acuity deteriorated from 20/20 to 20/80 postoperatively recovered to baseline within one month after surgery. Another patient experienced a decrease in visual acuity from 20/30 to hand movements immediately postoperatively and showed rapid improvement to 20/40 following high-dose intravenous methylprednisolone (1 g/day for 3 days), although a residual partial quadrantic visual field defect persisted.

Additional postoperative adverse events included mild ptosis in one patient and temporary periocular hypoesthesia in another, both of which resolved spontaneously or with conservative management. In our cohort, no severe adverse events such as postoperative optic disc atrophy, pupillary defects, or blindness were observed.

**Table 4:** Functional outcomes before and after surgery

Parameter	Preoperative	Postoperative
Mean Snellen visual acuity	0.86	0.87
Mean Hertel exophthalmometry	17.6 mm	15.9 mm
Diplopia	20 (33%)	9 (15%)
Visual acuity improved		24 (40%)
Visual acuity stable		34 (57%)
Visual acuity worsened		2 (3%)

## Discussion

Cryoextraction of orbital tumors was first described in 1985 [18] and has since been supported by numerous studies demonstrating its utility. Rosen et al. reported favorable outcomes in a large series of cryoextractions published in 2010 [16]. This study included 103 patients with various orbital tumors, of whom 30 had CVMs, representing the largest subgroup. Additional smaller retrospective cohort studies have reported the safety and effectiveness of cryoextraction for CVM removal, particularly in the setting of anterior transconjunctival orbitotomy [17, 19]. To our knowledge, no previous study has evaluated the impact of cryoextraction on surgical duration in anterior and lateral orbitotomy for orbital CVMs.

Due to variability in surgeon expertise and the wide range of available surgical approaches, cryoextraction has not been established as a standard procedure. Nevertheless, when appropriately indicated, it offers several advantages. For cryoextraction to be effective, the lesion should permit stable cryoprobe fixation and controlled traction during tumor delivery. Cavernous venous malformations often fulfill these conditions due to their encapsulated structure and characteristic tissue consistency.

In clinical practice, cryoextraction may be particularly advantageous for lesions located deep within the intraconal space, where direct manipulation is limited and controlled traction facilitates tumor delivery through a relatively small surgical corridor. Conversely, lesions with marked adhesions, irregular surfaces, friable consistency, or close attachment to critical neurovascular structures may be less suitable for cryo-assisted extraction, as excessive traction could increase the risk of tissue injury. In such situations, conventional stepwise blunt dissection may provide greater surgical control.

The demographic profile in our cohort aligns with existing literature, which reports a higher prevalence of CVMs in women and a typical onset in the fourth to fifth decade of life. Similarly, our anatomical distribution of 63% intraconal and 37% extraconal lesions is consistent with earlier series [2–5].

A cohort study of 164 patients by Strianese et al. elegantly illustrates the risk of postoperative complications following surgical excision of orbital CVMs, using both cryoextraction and conventional techniques [20]. Consistent with the findings of the present study, visual acuity remained stable or improved postoperatively in 99% of cases. Irreversible visual loss occurred in 2 patients (1%) immediately after surgery, and in both cases optic disc atrophy developed during follow-up. The presumed mechanism of vision loss was posterior optic nerve ischemia resulting from traction and manipulation of the optic nerve and surrounding tissues during tumor removal. Similarly, McNab reported 3 cases of permanent visual loss in a series of 85 patients and concluded that surgical risk increased for posteriorly located, long-standing tumors in close proximity to the optic nerve [4].

Postoperative improvement of diplopia was substantial in our cohort. Nevertheless, 2 patients (3%) required strabismus surgery and prismatic correction, both of whom had not undergone cryoextraction. Of the 30 patients who underwent cryoextraction, only 2 developed transient postoperative diplopia, which resolved spontaneously during follow-up. In the literature, postoperative diplopia has been reported in approximately 20% of cases, although it is temporary in most instances [15, 20].

While our findings demonstrate a statistically significant reduction in operative time with cryoextraction for the swinging eyelid approach (*p* = 0.04), this difference did not reach statistical significance in the lateral orbitotomy subgroup, likely due to the limited sample size. The retrospective design of the study may also introduce selection and information biases. Selection bias may also have influenced the observed differences between groups, as the choice of extraction technique was determined preoperatively according to radiological findings, anticipated surgical complexity, and evolving surgical practice throughout the study period rather than by random allocation. In addition, some functional ophthalmological parameters such as formal visual field testing and color vision assessment were not consistently documented throughout the entire study period due to the retrospective design and long inclusion interval. Nevertheless, our results suggest that cryoextraction may reduce operative time and postoperative complications in the surgical management of CVMs, thereby potentially improving patient recovery. To our knowledge, this is the first study to report an association between cryoextraction and reduced operative time in both anterior and lateral orbitotomy approaches for CVMs, providing comparative retrospective data in a field previously supported primarily by surgical experience.

## Conclusion

In this retrospective single-center series, cryoextraction was associated with shorter operative times and a favorable postoperative safety profile in the surgical management of orbital cavernous venous malformations. The reduction in surgery duration reached statistical significance for the transconjunctival anterior orbitotomy using the swinging eyelid approach and showed a consistent, albeit non-significant, trend in lateral orbitotomy. Importantly, postoperative complications—particularly persistent diplopia—occurred less frequently following cryo-assisted extraction, while visual outcomes remained excellent overall. These findings reinforce the technical advantages of cryoextraction in lesions with suitable anatomical characteristics, such as well-circumscribed cavernous venous malformations.

Although limited by its retrospective design and sample size, this study provides the first comparative evidence suggesting that cryoextraction may facilitate surgical efficiency without compromising safety across two commonly employed orbital approaches. When applied judiciously and in appropriately selected cases, cryoextraction represents a valuable technique in orbital surgery that can facilitate tumor delivery, minimize manipulation of surrounding structures, and potentially enhance postoperative recovery. Future prospective, multicenter studies are warranted to further validate these findings and to define standardized indications for cryo-assisted techniques in orbital tumor surgery.

## Data Availability

The datasets generated and/or analyzed during the current study are not publicly available due to institutional data protection regulations but are available from the corresponding author on reasonable request.

## Abbreviations

ADC: Apparent Diffusion Coefficient
CVM: Cavernous Venous Malformation
DWI: Diffusion–Weighted Imaging
MRI: Magnetic Resonance Imaging
OCH: Orbital Cavernous Hemangioma
